# Immunoglobulin-like transcript 4 promotes tumor progression and metastasis and up-regulates VEGF-C expression via ERK signaling pathway in non-small cell lung cancer

**DOI:** 10.18632/oncotarget.3624

**Published:** 2015-04-13

**Authors:** Pei Zhang, Xiaosun Guo, Juan Li, Shuwen Yu, Linlin Wang, Guosheng Jiang, Dong Yang, Zhaolong Wei, Nan Zhang, Jie Liu, Yuping Sun

**Affiliations:** ^1^ Department of Oncology, School of Medicine, Shandong University, Jinan, Shandong, P. R. China; ^2^ Department of Oncology, Jinan Central Hospital, Shandong University, Jinan, Shandong, P. R. China; ^3^ Department of Pathophysiology, School of Medicine, Shandong University, Jinan, Shandong, P. R. China; ^4^ Department of Pharmacy, Jinan Central Hospital, Shandong University, Jinan, Shandong, P. R. China; ^5^ Institute of Basic Medicine, Shandong Academy of Medical Sciences, Jinan, Shandong, P. R. China; ^6^ Department of Medical Imaging, Jinan Central Hospital, Shandong University, Jinan, Shandong, P. R. China

**Keywords:** ILT4, tumor progression, ERK1/2, VEGF-C, NSCLC

## Abstract

Immunoglobulin-like transcript (ILT) 4 has long been thought to be cell-surface molecule in certain immune cells and negatively regulates immune response. Recently, overexpression of ILT4 has been observed in a few cancers with unknown function. Here, we showed manipulation of ILT4 affected non-small cell lung cancer (NSCLC) cell proliferation, migration and invasion *in vitro* analyses. *In vivo*, ILT4 promoted the tumor growth and metastasis. Furthermore, the phosphorylation of extracellular regulated protein kinases (ERK1/2) was enhanced in ILT4 overexpressing NSCLC cells. ERK1/2 specific inhibitor U0126 suppressed the proliferation, migration and invasion of those cells. Stepwise investigations demonstrated that vascular endothelial growth factor C (VEGF-C) was the downstream effector of ILT4 and ERK1/2. Silence of VEGF-C attenuated the migration and invasion activity of ILT4 overexpressing cells. Moreover, Kaplan-Meier survival analysis indicated that NSCLC patients with ILT4 positive expression had a poor patient survival. ILT4 and VEGF-C expression had notable positive correlation in cancer cells, and their co-expression was significantly associated with adverse prognostic factors. Our findings suggest that ILT4 drives NSCLC development in part on activation of ERK signaling which in turn upregulates VEGF-C. ILT4 could be a novel cancer therapeutic target for NSCLC.

## INTRODUCTION

Lung cancer is one of the most common malignancies in the world and is also the leading reason of cancer deaths worldwide, causing 1.4 million deaths annually [1, 2]. Non-small cell lung cancer (NSCLC) is the primary histological type of lung cancer, accounting for about 85% of the disease [3]. Although gradual improvements in survival have been achieved, this advance has not matched those seen in other common malignancies, partly as a result of NSCLC patients often presenting at an advanced stage [4, 5]. Therefore, it is essential to further elucidate the underlying molecular mechanisms of NSCLC progression and metastasis, and to develop novel therapeutic approaches.

The immunoglobulin-like transcript (ILT) family is a group of cell-surface protein receptors preferentially expressed on myeloid lineage cells, and is divided into activating receptors (ILT1, ILT6, ILT7 and ILT8) and inhibitory receptors (ILT2, ILT3, ILT4 and ILT5) [6–10]. Inhibitory receptors possess long cytoplasmic tails that contain immunoreceptor tyrosine-based inhibitory motifs (ITIMs), which recruit protein tyrosine phosphatase SHP-1 to inhibit myeloid cell activation [7]. In addition, several studies indicate that inhibitory receptors ILT2, ILT3 and ILT4 are frequently up-regulated in certain myeloid malignant tumors and correlate with adverse prognosis [11–14]. ILT2 is found to express in some T-cell lymphomas, and ILT2 expressing Se'zary cells are resistant to CD3 monoclonal antibody induced cell death [11, 13]. ILT3 and ILT4 expression represent the phenotypic abnormality in chronic lymphocytic leukemia (CLL) B cells, and ILT3 expression is more common in CLL patients with lymphoid tissue involvement [12]. A recent study has demonstrated that ILT4 and its mouse ortholog paired Ig-like receptor (PIRB) promote haematopoietic stem cells (HSCs) repopulation and acute myeloid leukemia (AML) development [14].

Interestingly, ILT4, as well as other inhibitory receptors ILT2 and ILT3, has also been detected in a few non-myeloid malignant tumors cells [15–19]. *In vitro*, high expression of both ILT2 and ILT3 in gastric cancer cells inhibit cytotoxic activity of NK cells; and in primary human gastric cancer tissues, ILT2 correlates with poor cell differentiation and large tumor [16]. ILT3 expression is significantly associated with ovarian tumor progression in laying hen model of spontaneous ovarian cancer [15]. High ILT4 expression in NSCLC and breast cancer cells is associated with lymph node metastasis and less tumor infiltrated lymphocytes (TILs) [17, 18]. Recently, it is reported that higher ILT4 was demonstrated in more aggressive pancreatic ductal carcinoma cell lines [19]. All these results suggest that the aberrant expression of inhibitory ILTs including ILT4 may play an important role in tumor progression. However, the exact function of ILT4 in cancer malignancy, especially in non-myeloid malignant tumors, and the underlying molecular mechanism have been poorly understood.

In this study, we investigated the biological function of ILT4 in NSCLC. By manipulating ILT4 expression in NSCLC cells, we found ILT4 was involved in cell proliferation and motility *in vitro*, as well as tumor growth and metastasis *in vivo*. Furthermore, we observed ILT4 upregulated vascular endothelial growth factor C (VEGF-C) expression via activating extracellular regulated protein kinases (ERK1/2). Inhibition of either VEGF-C or ERK1/2 clearly attenuated ILT4-induced adverse phenotype. In addition, ILT4 expression was positively correlated with VEGF-C in primary human NSCLC tissues, and associated with poor prognosis. These results reveal that ILT4 functions as a tumor enhancer in NSCLC that promotes tumor growth and metastasis, at least partly, through activation of ERK signaling and upregulation of VEGF-C expression.

## RESULTS

### ILT4 promotes NSCLC cell proliferation and motility *in vitro*

**Figure 1:**
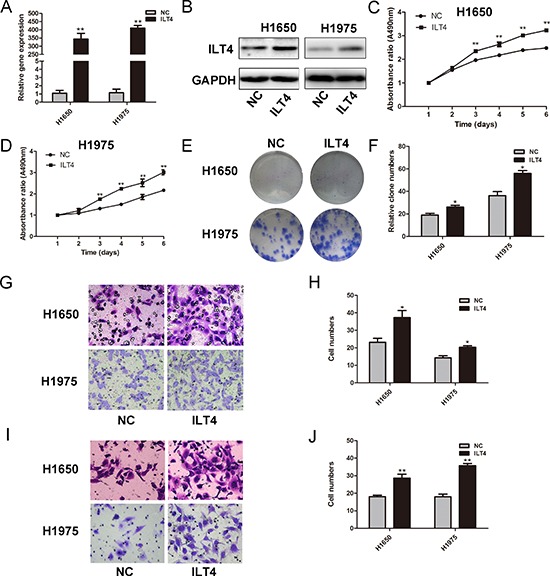
ILT4 significantly promotes cell proliferation and motility of NSCLC cell lines ILT4 expression in H1650 and H1975 cells transfected with ILT4 vector and empty vector by **A.** PCR and **B.** Western blot analysis. Comparison of cell proliferation of **C.** H1650 and **D.** H1975 cells transfected with ILT4 vector and empty vector by MTT assay. **E.** Comparison of colony formation of H1650 and H1975 cells transfected with ILT4 vector and empty vector by colony formation assay. **F.** The foci numbers of clones. Comparison of **G.** and **H.** cell migration and **I.** and **J.** invasion of H1650 and H1975 cells transfected with ILT4 vector and empty vector by the invasion and migration assay, respectively. (Magnification × 400) The error bars indicate ± SEM. *P < 0.05; **P < 0.01 by Student's *t*-test. All the results were repeated thrice.

To assess the potential role of ILT4 in NSCLC cells, we determined ILT4 expression in NSCLC cell lines (A549, H1299, H226, H1975 and H1650) (Supplementary Figure 1). Then, H1650 and H1975 cells with endogenous low ILT4 expression were selected to be transfected with ILT4 vector, leading to significant upregulation of ILT4 (Figure 1A and 1B). H1650 and H1975 cells transfected with ILT4 vector proliferated at a higher rate than those with corresponding vector transfection (Figure 1C and 1D), and their colony-formation ability was improved (Figure 1E and 1F and Supplementary Figure 2A). As well, the migration and invasion in H1650 and H1975 cells transfected with ILT4 vector were increased (Figure 1G–1J).

**Figure 2:**
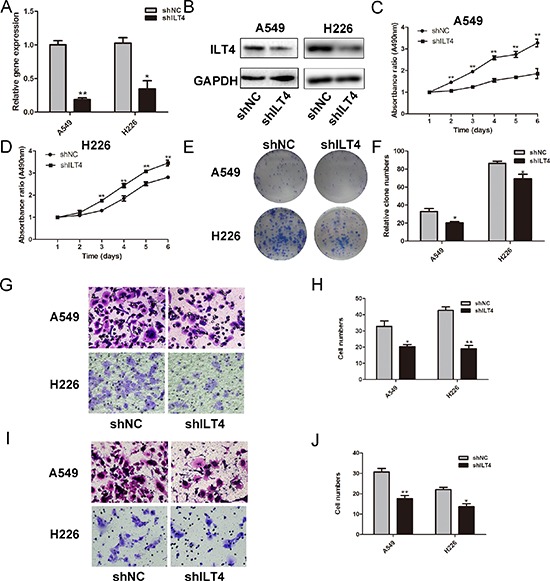
Knockdown of ILT4 inhibits cell proliferation and motility of NSCLC cell lines ILT4 expression in A549 and H226 cells transfected with shILT4 vector and empty vector by **A.** PCR and **B.** Western blot analysis. Comparison of cell proliferation of **C.** A549 and **D.** H226 cells transfected with shILT4 vector and empty vector by MTT assay. **E.** Comparison of colony formation of A549 and H226 cells transfected with shILT4 vector and empty vector by colony formation assay. **F.** The foci numbers of clones. Comparison of **G.** and **H.** cell migration **I.** and **J.** invasion of A549 and H226 cells transfected with shILT4 vector and empty vector by the invasion and migration assay, respectively. (Magnification × 400) The error bars indicate ± SEM. *P < 0.05; **P < 0.01 by Student's *t*-test. All the results were repeated thrice.

Next, ILT4 shRNA vector signifantly decreased ILT4 expression in A549 and H226 cells with endogenous high ILT4 expression (Figure 2A and 2B). Silence of ILT4 reduced cell proliferation rate and impaired the colony-formation ability (Figure 2C–2F and Supplementary Figure 2B). Meanwhile, the cell migration and invasion were also significantly decreased in A549 and H226 cells transfected with ILT4 shRNA vector (Figure 2G–2J). Thus, ILT4 promoted proliferation and motility of NSCLC cells.

Besides, we also determined whether ILT4 affected cell apoptosis or not. The apoptosis assay showed that both overexpression and underexpression of ILT4 did not influence cell apoptosis in NSCLC cells (Supplementary Figure 3).

### ILT4 drives NSCLC tumor growth and metastasis *in vivo*

**Figure 3:**
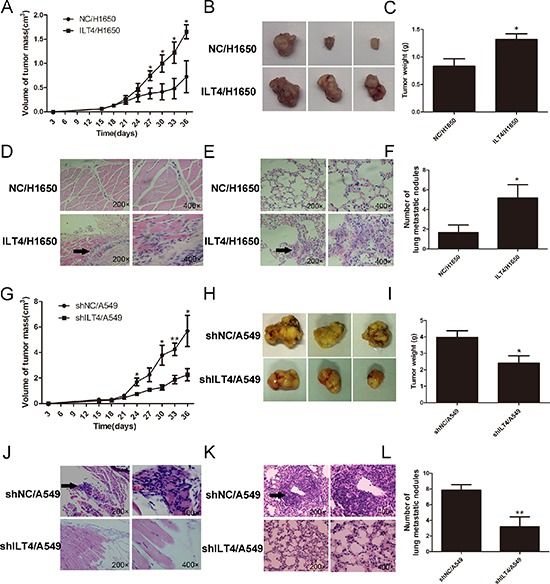
ILT4 drives NSCLC tumor growth and metastasis *in vivo* Comparison of **A.** and **B.** tumor size and **C.** tumor weight of mice after subcutaneous injection with ILT4/H1650 and NC/H1650 cells. **D.** Comparison of local invasion around the tumors, with islands of cancer cells invading the peritumoral muscle of mice after subcutaneous injection with ILT4/H1650 and NC/H1650 cells. Representative results of HE staining of the metastatic cells in the muscle. **E.** Comparison of lung metastasis in mice after intravenous injection with ILT4/H1650 and NC/H1650 cells. Representative results of HE staining of the metastatic nodules in the lung. **F.** Comparison of tumor macrometastatic nodules in the lungs of mice after intravenous injection with ILT4/H1650 and NC/H1650 cells. Comparison of **G.** and **H.** tumor size and **I.** tumor weight of mice after subcutaneous injection with shILT4/A549 and NC/A549 cells. **J.** Comparison of the islands of cancer cells invading the peritumoral muscle of mice after subcutaneous injection with shILT4/A549 and NC/A549 cells. **K.** Comparison of lung metastasis in mice after intravenous injection with shILT4/A549 and NC/A549 cells. **L.** Comparison of the number of tumor macrometastatic nodules in the lungs of mice after intravenous injection with shILT4/A549 and NC/A549 cells. *n* = 6. The error bars indicate ± SEM. *P < 0.05; **P < 0.01 by Student's *t*-test.

To address whether ILT4 promotes tumor growth *in vivo*, stable transfected ILT4/H1650 and shILT4/A549 cells and their corresponding control cells were implanted in left flank of the mice to form ectopic tumors. The tumor volumes were much massive in mice with injection of ILT4/H1650 cells and smaller in mice with injection of shILT4/A549 cells at the designated time points, compared with their corresponding control cells, respectively (Figure 3A and 3B, 3G and 3H). At day 36, compared with their corresponding control groups, the tumor weight in ILT4/H1650 group was increased and that in shILT4/A549 group was significantly decreased (Figure 3C and 3I). Interestingly, ILT4/H1650 cell invasion into peritumoral muscle layers was also enhanced. Meanwhile, the invasion of shILT4/A549 was inhibited, compared with their control groups (Figure 3D and 3J).

Next, to evaluate whether ILT4 promotes distant metastasis *in vivo*, mice were injected with ILT4/H1650 and shILT4/A549 and their corresponding control cells via the tail vein, respectively. At day 42, the number of lung macrometastases was much more in ILT4/H1650-treated mice and much less in shILT4/A549-treated mice, compared to their control groups (Figure 3E and 3F, 3K and 3L).

### ILT4 induces malignant phenotype of NSCLC cells through activating ERK signaling pathway

**Figure 4:**
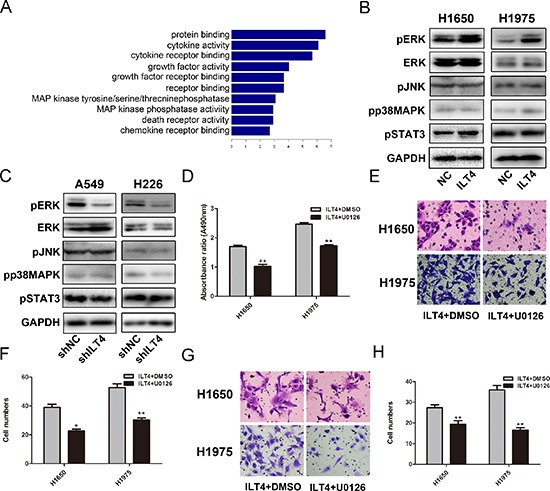
Activation of ERK1/2 involves in ILT4-driven NSCLC cell growth and motility **A.** Functional annotation of clustering of genes regulated by ILT4. Enriched groups are named by the gene ontology term of the group member with the most significant *P* value and are ranked by the groups enrichment score. **B.** The expression levels of pERK, ERK, pJNK, pp38MAPK, pSTAT3 and GAPDH in H1650 and H1975 cells transfected with ILT4 vector and empty vector by Western blot analysis. **C.** The expression levels of pERK, ERK, pJNK, pp38MAPK, pSTAT3 and GAPDH in A549 and H226 cells transfected with shILT4 vector and empty vector cells by Western blot analysis. The **D.** cell proliferation and **E.** and **F.** cell migration and **G.** and **H.** invasion advantages of ILT4 overexpressing H1650 and H1975 cells after inhibiting ERK activation by U0126 (30nM). (Magnification × 400) The error bars indicate ± SEM. *P < 0.05; **P < 0.01 by Student's *t*-test. All the results were repeated thrice.

To investigate the intrinic mechanisms by which ILT4 promotes cell proliferation and motility, we examined the effect of ILT4 overexpression on gene expression profiles. Functional annotation displayed the change of multiple genes related to MAP kinase (MAPK) phosphatase activity in ILT4 overexpressing H1650 cells (Figure 4A). Then, we detected the phosphorylation of three parallel signal transduction modules, including JNK, p38, and ERK, in MAPK signaling and found the phosphorylation of ERK1/2 was significantly enhanced in ILT4 overexpressing NSCLC cells (Figure 4B) and inhibited in ILT4 knockdown cells (Figure 4C).

Activation of ERK signaling pathway enhances cancer cell growth and metastasis in NSCLC cells, we next investigated whether ILT4 promotes cell malignant phenotype through activating ERK signal pathway. ERK1/2 inhibitor U0126 was used to treat ILT4 overexpressing cells. The proliferation, migration and invasion ability of those cells were significantly suppressed (Figure 4D–4H).

### ILT4 upregulates VEGF-C expression via ERK signal pathway

**Figure 5:**
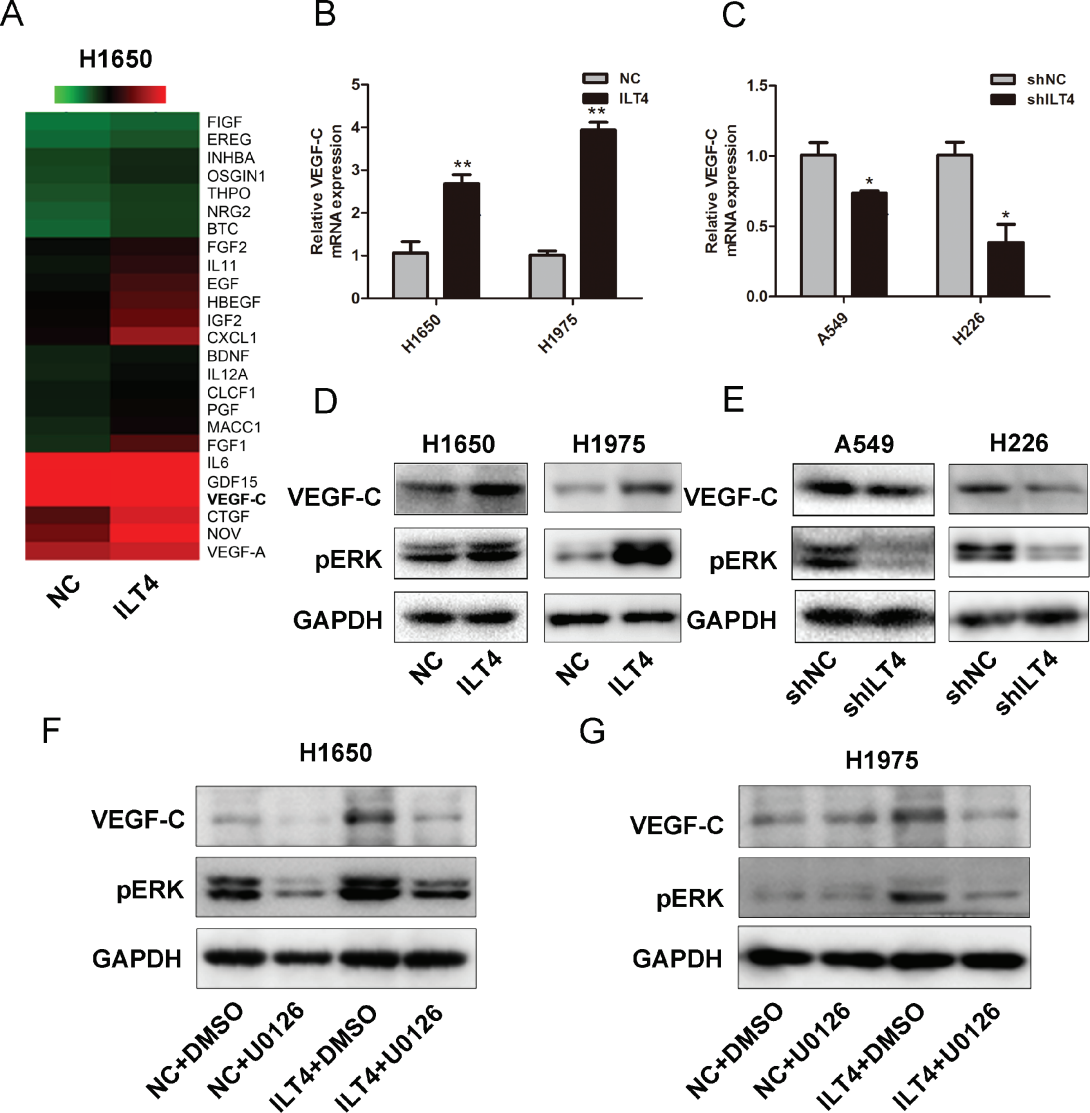
ILT4 up-regulates VEGF-C expression through ERK1/2 activation in NSCLC cells **A.** Gene expression levels for the subset of growth factors affected by ILT4. Red, high expression; green, low expression. VEGF-C expression level in H1650 and H1975 cells transfected with ILT4 vector and empty vector by **B.** PCR and **D.** Western blot analysis. VEGF-C expression level in A549 and H226 cells transfected with shILT4 vector and the empty vector by **C.** PCR and **E.** Western blot analysis. VEGF-C expression level in ILT4 overexpressing H1650 and H1975 cells after treated with ERK inhibitor U0126 (30nM) by Western blot analysis **F.** and **G.** The error bars indicate ± SEM. *P < 0.05; **P < 0.01 by Student's *t*-test. All the results were repeated thrice.

As shown in Figure 4A, the functional annotation also displayed the change of multiple genes related to growth factor activity in ILT4 overexpressing H1650 cells. And hot map showed a cluster of growth factor genes including VEGF-C were upregulated in those cells (Figure 5A). Next, using qRT-PCR and Western blot, we confirmed the mRNA and protein levels of VEGF-C were elevated in ILT4 overexpressing cells (Figure 5B and 5D), and decreased in ILT4 knockdown cells (Figure 5C and 5E).

In addition, as ERK signaling pathway affects VEGF-C expression in tumor cells [20, 21], we examined whether ERK signal participates in ILT4-induced VEGF-C expression. H1650 and H1795 cells were treated with both ILT4 vector and ERK1/2 inhibitor-U0126. The ILT4 overexpression-induced VEGF-C level was efficiently attenuated by U0126 treatment (Figure 5F and 5G). Therefore, induction of VEGF-C expression in ILT4 overexpressing H1650 and H1795 cells depends at least in part on the acitvation of ERK signaling by ILT4.

### ILT4 induces malignant phenotype of NSCLC cells through VEGF-C

**Figure 6:**
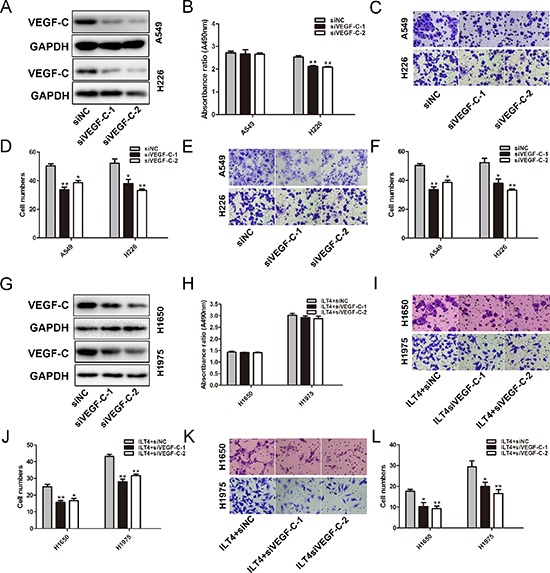
VEGF-C involves in ILT4-driven NSCLC cell migration and invasion **A.** VEGF-C expression in A549 and H226 cells transfected with siVEGF-C and negative control siRNA by Western blot analysis. The **B.** cell proliferation and **C.** and **D.** cell migration and **E.** and **F.** invasion of A549 and H226 cells cells after transfected with siVEGF-C. **G.** VEGF-C expression in ILT4 overexpressing H1650 and H1975 cells transfected with siVEGF-C and negative control siRNA by Western blot analysis. The **H.** cell proliferation and **I.** and **J.** cell migration and **K.** and **L.** invasion of ILT4 overexpressing H1650 and H1975 cells after transfected with siVEGF-C. (Magnification × 400) The error bars indicate ± SEM. *P < 0.05; **P < 0.01 by Student's *t*-test. All the results were repeated thrice.

Since VEGF-C is crucial for NSCLC cell growth and metastasis [22], we investigated whether ILT4 enhances NSCLC cell malignant phenotypes via upregulating VEGF-C expression. Firstly, we determined the function of VEGF-C in NSCLC cells, A549 and H226 cells with endogenous high ILT4 expression were selected to be transfected with siVEGF-C (Figure 6A). Although the cell proliferation was not significantly attenuated in A549 cell transfected with siVEGF-C (Figure 6B), VEGF-C siRNA (siVEGF-C) could inhibit cell proliferation in H226 cells (Figure 6B). In addition, knockdown of VEGF-C expression significantly inhibited the migration and invasion of A549 and H226 cells (Figure 6C–6F).

Furthermore, ILT4 overexpressing H1650 and H1975 cells were transfected with siVEGF-C, leading to the downregulation of VEGF-C (Figure 6G). And then the phenotypic changes of cell proliferation, migration and invasion were analyzed. Knockdown of VEGF-C inhibited the migratory and invasive behaviors of ILT4 over-expressing H1650 and H1975 cells (Figure 6I–6L). However, VEGF-C downregulation in those cells had no effect on cell proliferation (Figure 6H).

### ILT4 and its co-expression with VEGF-C in human NSCLC tissues

**Figure 7:**
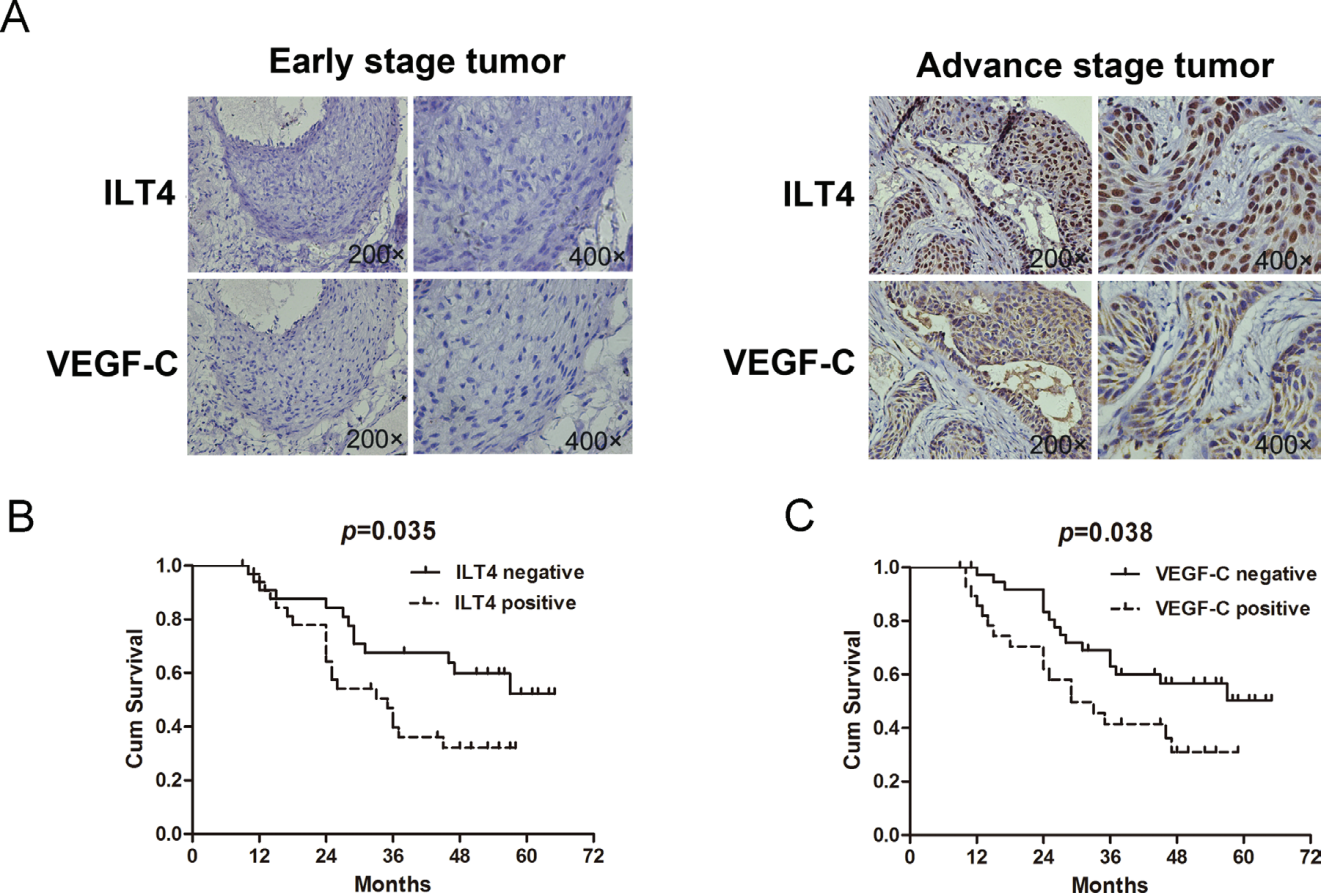
Co-expression of ILT4 and VEGF-C in NSCLC tissues **A.** Co-expression of ILT4 and VEGF-C in tumor specimens. **B.** Survival analysis of NSCLC patients with or without ILT4 expression by Kaplan-Meier survival analysis. (Long-rank test) **C.** Survival analysis of NSCLC patients with or without VEGF-C expression. (Long-rank test).

Using immunohistochemical staining, we determined ILT4 and VEGF-C expression in 105 primary human NSCLC tissues, and analyzed their associations with clinicopathological parameters and patient survival time. The expression of ILT4 was displayed in the cancer cell nucleus, membrane or/and cytoplasm (Supplementary Figure 4), and ILT4 expressing tissues accounted for 45.7% (48/105) of the whole samples. In adjacent normal lung tissues, ILT4 staining is too weak or not observed (Data not shown). Conversely, a significant overexpression of ILT4 was observed in tissues with advance stage (Figure 7A). Positive ILT4 expression in cancer cells was correlated with worse cell differentiation (*P* = 0.038), regional lymph node involvement (*P* = 0.04), advanced stages (*P* = 0.013), and age of more than 60 years (*P* = 0.044). (Supplementary Table 1).

In addition, we observed the expression pattern of ILT4 was consistent with that of VEGF-C (Figure 7A and Supplementary Figure 5). Moreover, co-expression of ILT4 and VEGF-C (ILT4+/VEGF-C+) was significantly associated with regional lymph node involvement (*P* = 0.008) and advanced stages (*P* = 0.002) compared with double negative group (ILT4−/VEGF-C−). Also, their co-expression was related to female gender (*P* = 0.025), smoking history of more than 30 years (*P* = 0.025) and worse cell differentiation (*P* = 0.012) compared with VEGF-C positive expression alone (ILT4-/VEGF-C+), and correlated with squamous NSCLC (*P* = 0.013) compared with ILT4 positive expression alone (ILT4+/VEGF-C-). (Supplementary Table 2).

Importantly, we examined the prognosis significance of ILT4 and VEGF-C in NSCLC patients. Kaplan-Meier analysis showed that the overall survival (OS) of ILT4 and VEGF-C expressing group was lower than the corresponding negative group, respectively (Figure 7B and 7C, ILT4, *P* = 0.035; VEGF-C, *P* = 0.038). In addition, the OS of patients with ILT4+VEGF-C+ was much lower than that of group with ILT4−/VEGF-C− (Supplemetary Figure 6A, *P* = 0.009), but not than that of group with ILT4-/VEGF-C+ or ILT4+/VEGF-C- (Supplemetary Figure 6B and 6C, ILT4-/VEGF-C+, *P* = 0.741; ILT4+/VEGF-C-, *P* = 0.501).

## DISCUSSION

ILT4 is mainly expressed in myeloid lineage cells, and most studies focus on the role of ILT4 on DCs and identify ILT4 as an inhibitory biomarker of DCs [23–26]. Recently, it is demonstrated that ILT4 high expression has been found in leukemia. In mouse transplantation AML models, ILT4 ortholog PIRB inhibits the differentiation of leukemia cells, leading to AML development [14]. Our previous studies also found overexpression of ILT4 in breast cancer and NSCLC cells. However, the exact function of ILT4 in cancer has remained unclear. Here, we provided evidences that ILT4 promoted tumor growth and metastasis in NSCLC. *In vitro* analyses of manipulating ILT4 expression suggested that ILT4 dramatically enhanced cell proliferation, migration and invasion. *In vivo* assays further demonstrated ILT4 functioned in tumor growth, local invasion and distant metastasis. Importantly, high ILT4 expression was more frequently observed in NSCLC patients with adverse clinical parameters and low OS, indicating ILT4 was a poor prognostic factor in NSCLC patients. Taken together, we conclude that ILT4 is involved in the pathogenesis of NSCLC through promoting tumor cell growth and metastasis.

Also, the potential mechanisms of ILT4 in tumor progression were investigated. We found that ILT4 markedly activated ERK signaling pathway. ERK signaling pathway is one of the best-characterized kinase cascades in cancer cell biology and plays a central role in the carcinogenesis and maintenance of cancer [27–30]. In NSCLC, ERK signal is critical in cell differentiation, proliferation, survival, migration, and angiogenesis [31, 32]. In our study, the phosphorylation of ERK1/2 was found to be elevated in ILT4 overexpressing NSCLC cells. After treatment with ERK1/2 selective inhibitor (U0126), the proliferation and motility of those cells were decreased, supporting that ILT4 induces cancer cell malignant phenotype in NSCLC by activating ERK signaling pathway.

In addition, we found VEGF-C expression was increased in ILT4 overexpressing NSCLC cells. VEGF-C belongs to the vascular endothelial growth factor family and participates in tumor progression of human cancers including NSCLC. At present, accumulating data have indicated that VEGF-C synthesized in cancer cells promotes tumor progression via enhancing cell proliferation, invasion and metastasis [22, 33–36]. Moreover, it is reported that several immune-associated molecules highjacked by tumor cells lead to VEGF-C expression and increased tumor growth and metastasis [37, 38]. Consisted with the studies, here, we found knock-down of VEGF-C in H1650 cells transfected with ILT4 vector inhibited the incentive effects of ILT4 on cell migration and invasion. Since various NSCLC cell lines, including H1650, H1975, A549 and H226 cells we used, express vascular endothelial growth factor receptor (VEGFR) [39–41], we concluded that increased VEGF-C by overexpressing ILT4 might enhance tumor cells motility by binding its receptor. However, VEGF-C depletion did not influence the proliferation in those cells. The reason might be the modulation of other cellular pathways or proteins induced by ILT4 compensated for the inhibition of cell growth mediated by VEGF-C downregulation and the difference among the cell properties. Significantly, a positive correlation between ILT4 and VEGF-C expression was observed in NSCLC tumor tissues and their co-expression was related to adverse clinicpathological features and short survival. Collectively, our results suggest that ILT4 promotes tumor progression and metastasis, at least partly, through up-regulating VEGF-C expression in NSCLC.

As we know, ERK1/2 activation influences the expression of many growth factors in cancer cells including VEGF-C [20, 21, 42]. Since ILT4 enhanced ERK signaling and increased VEGF-C expression, we supposed that ILT4 overexpression upregulated VEGF-C expression by activating ERK signals in NSCLC cells. Actually, our results showed that inhibition of ERK1/2 reduced the expression of VEGF-C in NSCLC cells, which confirmed our hypothesis. However, we failed to identify the transcription factors which was activated by ILT4 and directly modulated VEGF-C transcription in the ERK signaling; thus, further research using co-immunoprecipitation and direct binding assays will be necessary.

In this study, overexpression of ILT4 alone could promote NSCLC cells proliferation, migration and invasion. Besides that, ILT4 was expressed in the membrane and cytoplasm, as well as in the cell nucleus, which was different with our previous study [18]. We identified this finding using two kinds of antibody known to specifically recognize ILT4. And the nuclear localization of ILT4 was also confirmed by Western blot and immunofluorescence in NSCLC cell lines (Supplementary Figure 7). The difference could be explained either by the promotion of primary antibody production, or by more effective enhanced primer and secondary antibody. Moreover, in order to provide more clues about whether membrane or intracellular ILT4 played a role in NSCLC development, we used the anti-ILT4 blocking antibody treatment in NSCLC cells and found blocking ILT4 could slightly inhibit the aggressive phenotype in NSCLC cells, but not change the expressional level of VEGF-C (Supplementary Figure 8). It seems that different location of ILT4 in NSCLC cells might promote tumor progression through different patterns, which need further deeper study.

In summary, our study demonstrates that ILT4 plays an important role in promoting tumor growth and metastasis, and activation of ILT4-ERK-VEGF-C axis may mediate tumor progression. Moreover, ILT4 can function as a useful biomarker to predict the prognosis of NSCLC patients. Therefore, targeting ILT4 may be a feasible and effective approach for NSCLC treatment.

## MATERIALS AND METHODS

### Patients

A total of 105 specimens were obtained from NSCLC patients admitted to Jinan Central Hospital of Shandong University from 2008 to 2013. There were 77 men (73.33%) and 28 women (26.67%) with median age of 62 years (range: 35-82 years) at the time of diagnosis. All patients received no preoperative therapy before sample collection. Patients from 2008 to 2010 were contacted by phone to check upon their health status and the last censor date was on August 30th, 2014. The stage of NSCLC was categorized according to surgical and pathological findings, which were based on the guidelines described by the sixth edition of AJCC/UICC. Written informed consents were obtained from each patient. The study was approved by the Institutional Review Board of Jinan Central Hospital of Shandong University.

### Cell culture

The human NSCLC cell lines (H226, H1299, H1650, H1975 and A549) were purchased from the cell resource center of Chinese Academy of Sciences (Beijing, China). H226, H1299, H1650 and H1975 cells were cultured in RPMI-1640 medium (Hyclone, Logan, UT, USA) supplemented with 10% fetal bovine serum (FBS; Hyclone, Logan, UT, USA); A549 cells were cultured in DMEM medium (Hyclone) supplemented with 10% FBS (Hyclone) ERK1/2 inhibitor U0126 was purchased from commercial source (Selleck, Houston, TX, USA).

### Cell transfection

ILT4 vector (GeneCopoeia, Rockville, MD, USA) was formed using full length human ILT4 cDNA linked with the Pez-lv105 vector to induce ILT4 over-expression in the cultured cells. ILT4 vector was transfected into the cells using X-treme GENE HP Reagents (Roche, Basel, Switzerland) according to the manufacturer's instructions. Cells transfected with Pez-lv105 (NC) vector served as negative control. Human ILT4 linked with pLenti6.3 vector (Invitrogen, Carlsbad, CA, USA) was used to induce ILT4 overexpressing in the cell lines. The stable cell lines were selected by blasticidin (Invitrogen) containing medium for 48h after transfection. The resulting stably transfected cell lines were collected after 6 weeks.

Human ILT4 shRNA linked with Lentivirus pGLV/H1/GFP vector (Genechem Co., Ltd., Shanghai, China) was used to induce ILT4-silence in the cell lines. Cells transfected with Lentivirus pGLV/H1/GFP-NC vector served as negative control. Stable cell lines were selected by incubating on puromycin (Invitrogen) containing medium for 48h after transfection. The resulting stably transfected cell lines were collected after 6 weeks. shRNA sequences are: shILT4, 5′-GAAGAAGAACACCCACAATGC-3′; shNC, 5′-GTTCTCCGAACGTGTCACGT-3′.

In order to silence VEGF-C expression, small interfering RNA against human VEGF-C (siVEGF-C) (Invitrogen) was transfected within NSCLC cells. siRNA sequences are: siVEGF-C-1,5′-AGGACAGAA GAGACUAUAATT-3′;5′-UUAUAGUCUCUUCUGUCC UTT-3′; siVEGF-C-2,5′-GGCUUAUGCAAGCAAAG AUTT-3′; 5′-AUCUUUGCUUGCAUAAGCCTT-3′; the contol (siNC), 5′-UUCUCCGAACGUGUCAC GUTT-3′; 5′-ACGUGACACGUUCGGAGA ATT-3′.

### Cell proliferation assay *in vitro*

The cell proliferation was determined using 3-(4,5-dimethylthiazole-2-yl)- 2,5-biphenyl tetrazolium bromide (MTT) (Solarbio, Beijing, China) assay. Cells were plated in 96-well plates at a started number of 2-4 × 10^3^ cells/well. The absorbance of each sample was measured at 490 nm for 6 days. The ratio of optical density (OD) value of each cell group normalised to the cells on Day 1 at the indicated time points are presented. Each experiment was performed in triplicate.

### Colony formation assay *in vitro*

The colony formation was determined using colony formation assay. Cells were plated in 6-well plates at a started number of 2-3 × 10^2^ cells. Cell colonies were stained with Giemsa (Solarbio) and counted after 2-3 weeks of culture. Each experiment was performed in triplicate.

### Cell migration assay and cell invasion assay *in vitro*

Cell migration was measured using 24-well transwell plates (Corning Incorporated, Corning, NY, USA) with 8μm-pore polycarbonate membranes. Matrigel invasion assay was done using membranes coated with matrigel matrix (BD Science, Sparks, MD, USA). Cells (2 × 10^5^ cell/ml) suspended in serum-free medium containing 0.1% BSA were added to the upper chamber and incubated for 12-24h at 37°C for migration assay and 24-30h for invasion assay. Migrated and Invasive cells were fixed with 100% methanol for 15 min and counted under a light optic microscope (Nikon) from 5 representative fields. Each experiment was performed in triplicate.

### Proliferation assays and metastasis assays *in vivo*

Male BALB/c nu/nu mice (4-6 weeks) were purchased from Beijing HFK Bioscience (Beijing, China). To induce ectopic tumor, 2 × 10^6^ cells were implanted in left flank of the mice (*n* = 6). The volume of tumor nodules was measured twice weekly. Tumor volume (V) was calculated as V = (a × b^2^)/2, where “a” and “b” are the long and short axis of the tumor nodule, respectively. After 36 days, tumor nodules were surgically excised and weighed. Histological analyses were used to detect local invasion in muscle layer in proximity to tumor nodules by hematoxylin-eosin staining (H&E) staining using microscope (Nikon, Tokyo, Japan).

To observe the role of ILT4 to distant metastasis, mice were injected with 5 × 10^5^ cells via tail vein. The mice were sacrificed and lungs were harvested at 42 days after injection. Visible lung surface macrometastatic white spots were counted using a dissecting microscope (Nikon). Histological analyses were used to detect metastasis in lungs which were embedded in paraffin and dyed by H&E.

Animal studies were conducted in accordance with the NIH animal use guidelines and current Chinese regulations and standards for laboratory animal use.

### Whole genome DNA microarray

NSCLC cells were harvested using total RNA isolation reagent TRIzol (Invitrogen) according to manufacturer's instructions. The samples were amplified and labeled using the Agilent Quick Amp labeling kit, and hybridized using the Whole Human Genome Oligo Microarray (Agilent Technologies, Palo Alto, CA, USA) using Agilent SureHyb hybridization chambers. After hybridization and washing, the processed slides were scanned with the Agilent DNA microarray scanner using the settings recommended. The data were collected using the Agilent Feature Extraction Software (version 11.0.1.1), and analyzed using GeneSpring GX software (version 11.5.1).

### qRT-PCR

NSCLC cells were harvest and lyzed in TRIzol (Invitrogen). First strand complementary DNAs were prepared using the First Strand cDNA Synthesis Kit (Fermentas, Burlington, Ontario, Canada). The qRT-PCR was carried out using cDNA as a template and Universal PCR Master Mix (Thermo Fisher Scientific, Waltham, MA, USA) on a CFX96 Touch Deep Well™ Real-Time PCR Detection System (Bio-Rad, Hercules, CA, USA). The amplification results for qRT-PCR was calculated using the 2(−ΔΔCt) method.

PCR reaction was performed using ILT4 primers: 5′-GCATCTTGGATTACACGGATACG-3′ (forward), 5′-CTGACAGCCATATCGCCCTG-3′(reverse); VEGF-C primers: 5′-TGGCAACATAACAGAGAACAGG-3′ (forward), and 5′- CAAACTCCTTCCCCACATCTAT-3′(reverse); GAPDH primers: 5′-AGAAGGCTGGGG CTCATTTG-3′ (forward), and 5′-AGGGGCCATCCA CAGTCTTC-3′(reverse).

### Western blot assay

The protein was separated by 10% SDS–PAGE gel and transferred onto a polyvinylidene difluoride membrane (Millipore, Boston, MA, USA). Subsequently, the membrane was blocked and incubated overnight at 4°C with the primary antibody including anti-ILT4 mAb (1: 400, Abgent, San Diego, CA, USA), anti-p-ERK pAb (1:1000, Epitomics, Burlingame, CA, USA), anti-ERK pAb (1:1000, Cell Signaling Technology, Danvers, MA, USA), anti-p-STAT3 pAb (1:1000, Epitomics), anti-JNK pAb (1:1000, Sangon Biotech, Shanghai, China), anti-p-p38MAPK pAb (1:1000, Sangon Biotech, Shanghai, China), and anti-VEGF-C pAb (1:1000, Abgent). Afterwards, the blots were labeled for 1 h with HRP-conjugated secondary antibodies (1:5000, Proteintech Group, Inc., Wuhan, China). Finally, the blots were exposed to the ChemiDoc™ XRS+ system (Bio-Rad, Hercules, CA, USA). The same membrane was probed for GAPDH (1:10000, Proteintech Group, Inc) as loading control.

### Immunohistochemistry

Paraffin sections were cut to a thickness of 4 μm and mounted on silanized slides. For NSCLC tissues, antigen retrieval with a microwave oven at 95°C for 10 min was applied before incubation with primary antibody. Subsequently, the sections were incubated with the primary antibodies against anti–ILT4 mAb (1:200, Abgent) (1:200, R&D Systems, Abingdon, UK) or anti-VEGF-C pAb (1:100, Abgent). Sections incubated with normal mouse or rabbit IgG instead of primary antibodies were used as negative control. After washing with PBS again, the sections were incubated with enhanced primer and HRP goat anti-mouse / rabbit IgG polymer by using Elivision™ plus Polyer HRP (Mouse/Rabbit) IHC Kit (Maixin Biotechnology Development Co., Ltd, Fuzhou, China). In turn, the sections were visualized by incubation with 3,3-diaminobenzidine solution (Maixin Biotechnology Development Co., Ltd). The nucleus was counterstained with hematoxylin.

Immunohistochemical analysis was performed by two independent investigators simultaneously. The percentage of stained cells was recorded by counting at least 5 random fields at a magnification of 400×. The proportion score represented the estimated fraction of positive staining tumor cell (0 = none; 1 = less than 25%; 2 = 25-75%; 3 = greater than 75%). The intensity score represented the estimated average staining intensity of positive tumor cell (0 = none; 1 = weak; 2 = intermediate; 3 = strong). The overall amount of protein present in each tumor was then expressed as the product or total score of the proportion and intensity scores for negative and positive tumor cells (ranges = 0-9, respectively). Cut point for positive: Score ≥ 4; cut point for negative: Score < 4.

### Statistical analysis

SPSS version 19.0 (SPSS, Chicago, IL, USA) was used for statistical analysis. Data were represented as mean ± standard deviation (SD), or mean ± standard error of the mean (SEM). The associations between the expression of ILT4/VEGF-C and clinicopathological variables were analyzed using Pearson's chi-squared test and the Student two-tailed *t* test. Overall survival time was determined as the duration from the date of initial diagnosis to death or to the last day of follow-up evaluation. Survival curves were drawn using the Kaplan-Meier method and compared by means of the log-rank test. *P* < 0.05 was considered statistically significant.

## SUPPLEMENTARY FIGURES AND TABLES


