# Supplementary material for: Immunoglobulin-like transcript 4 promotes tumor progression and metastasis and up-regulates VEGF-C expression via ERK signaling pathway in non-small cell lung cancer
Source: Oncotarget. 2015 Apr 13;6(15):13550–63. doi: 10.18632/oncotarget.3624 (PMC4537033; doi:10.18632/oncotarget.3624)
Supplement: Supplementary file 1 [file oncotarget-06-13550-s001.pdf]

SUPPLEMENTARY FIGURES AND TABLES

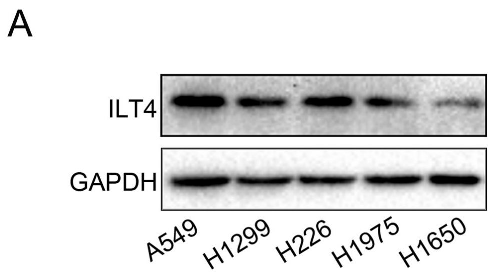

Supplementary Figure 1: The expression of ILT4 in NSCLC cell lines. A. ILT4 expression in NSCLC cell lines A549, H1299, H226, H1975 and H1650 by Western blot analysis.

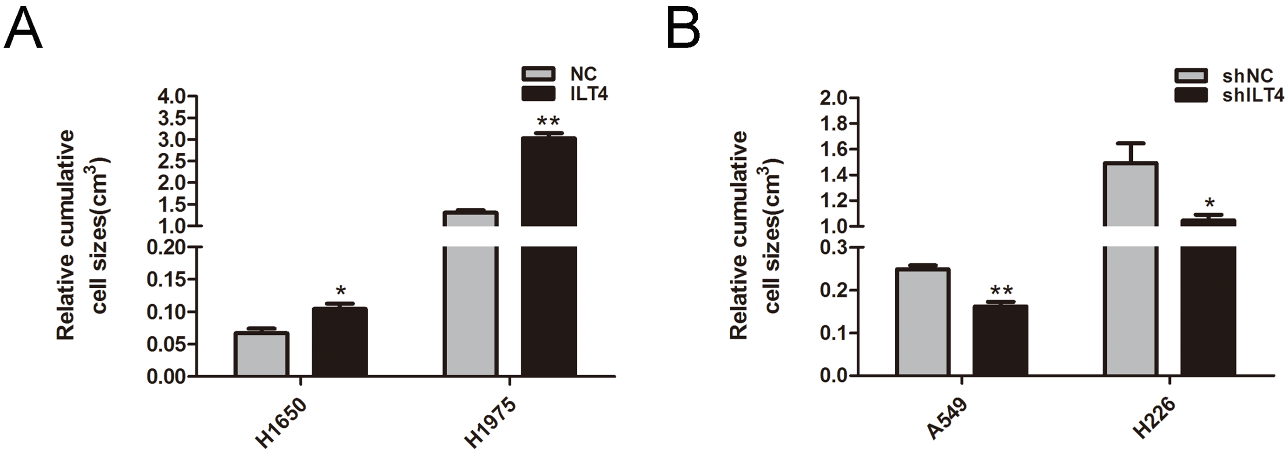

Supplementary Figure 2: The function of ILT4 on size of formed clones in NSCLC cell lines. A. Comparison of cumulative cell sizes of H1650 and H1975 cells transfected with ILT4 vector and empty vector. **B.** Comparison of cumulative cell sizes of A549 and H226 cells transfected with shILT4 vector and empty vector. The error bars indicate ± SEM. *$P < 0.05$; **$P < 0.01$ by Student's *t*-test. All the results were repeated thrice.

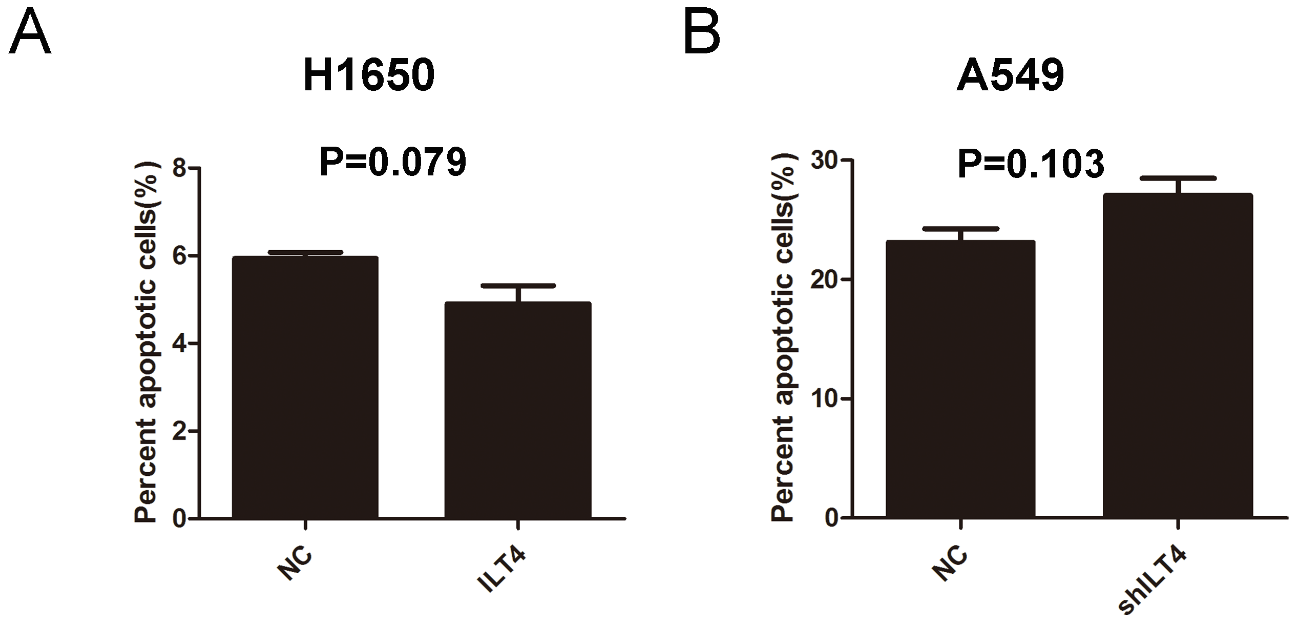

Supplementary Figure 3: The function of ILT4 on cell apoptosis of NSCLC cell lines. A. Comparison of cell apoptosis of H1650 cells transfected with ILT4 vector and empty vector by Annexin V-FITC/PI assay. **B.** Comparison of cell apoptosis of A549 cells transfected with shILT4 vector and empty vector by Annexin V-FITC/PI assay. The error bars indicate ± SEM. *$P < 0.05$; **$P < 0.01$ by Student's *t*-test. All the results were repeated thrice.

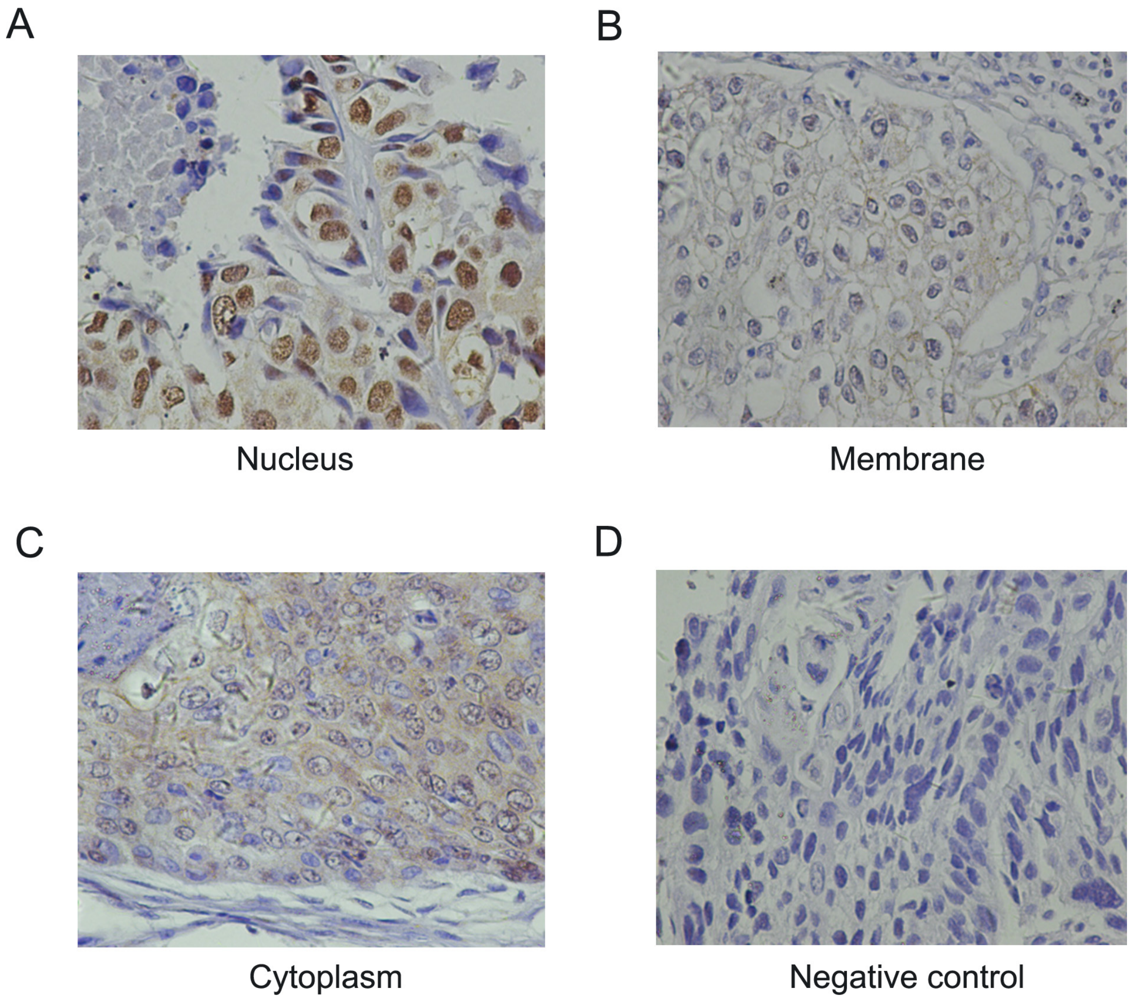

Supplementary Figure 4: The expression of ILT4 in human NSCLC tissues. A. ILT4 was predominantly localized in the nucleus. **B.** ILT4 was predominantly localized on the membrane. **C.** ILT4 was predominantly expressed in the cytoplasm. **D.** Negative control.(Magnification × 400).

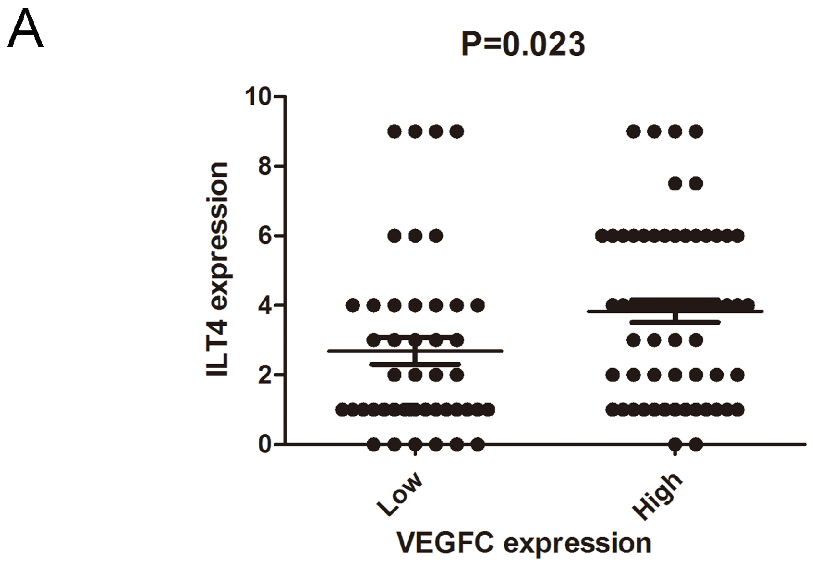

Supplementary Figure 5: Co-expression of ILT4 and VEGF-C in NSCLC tissues. A. ILT4 expression density between VEGF-C positive and negative group. (Student's *t* test).

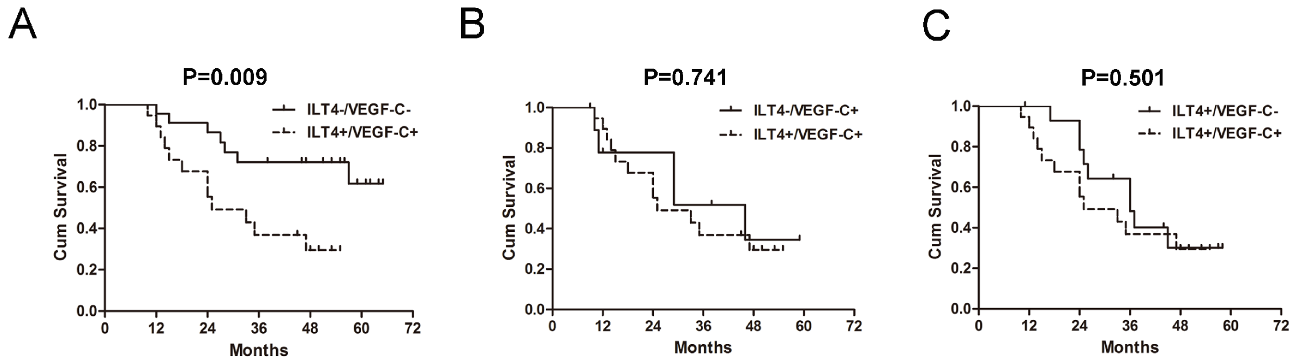

Supplementary Figure 6: Relationship between ILT4+/VEGF-C+ expression and patient survival. A. Survival analysis of NSCLC patients with ILT4+/VEGF-C+ expression and ILT4-/VEGF-C- expression. (Long-rank test) **B.** Survival analysis of NSCLC patients with ILT4+/VEGF-C+ expression and ILT4-/VEGF-C+ expression. (Long-rank test) **C.** Survival analysis of NSCLC patients with ILT4+/VEGF-C+ expression and ILT4+/VEGF-C- expression. (Long-rank test).

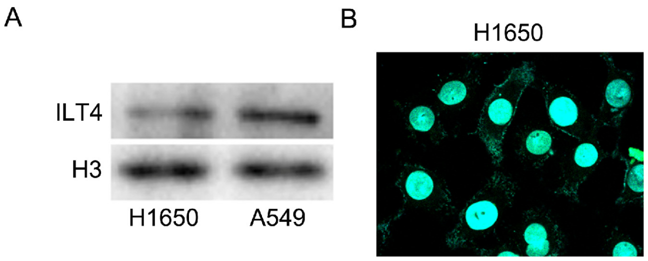

Supplementary Figure 7: The nuclear expression of ILT4 in human NSCLC cells. A. ILT4 and Histone H3 (nuclear marker) protein was detected in nuclear fraction of whole cell lysate. **B.** The expressional pattern of ILT4 in human NSCLC H1650 cell. (Magnification × 630)

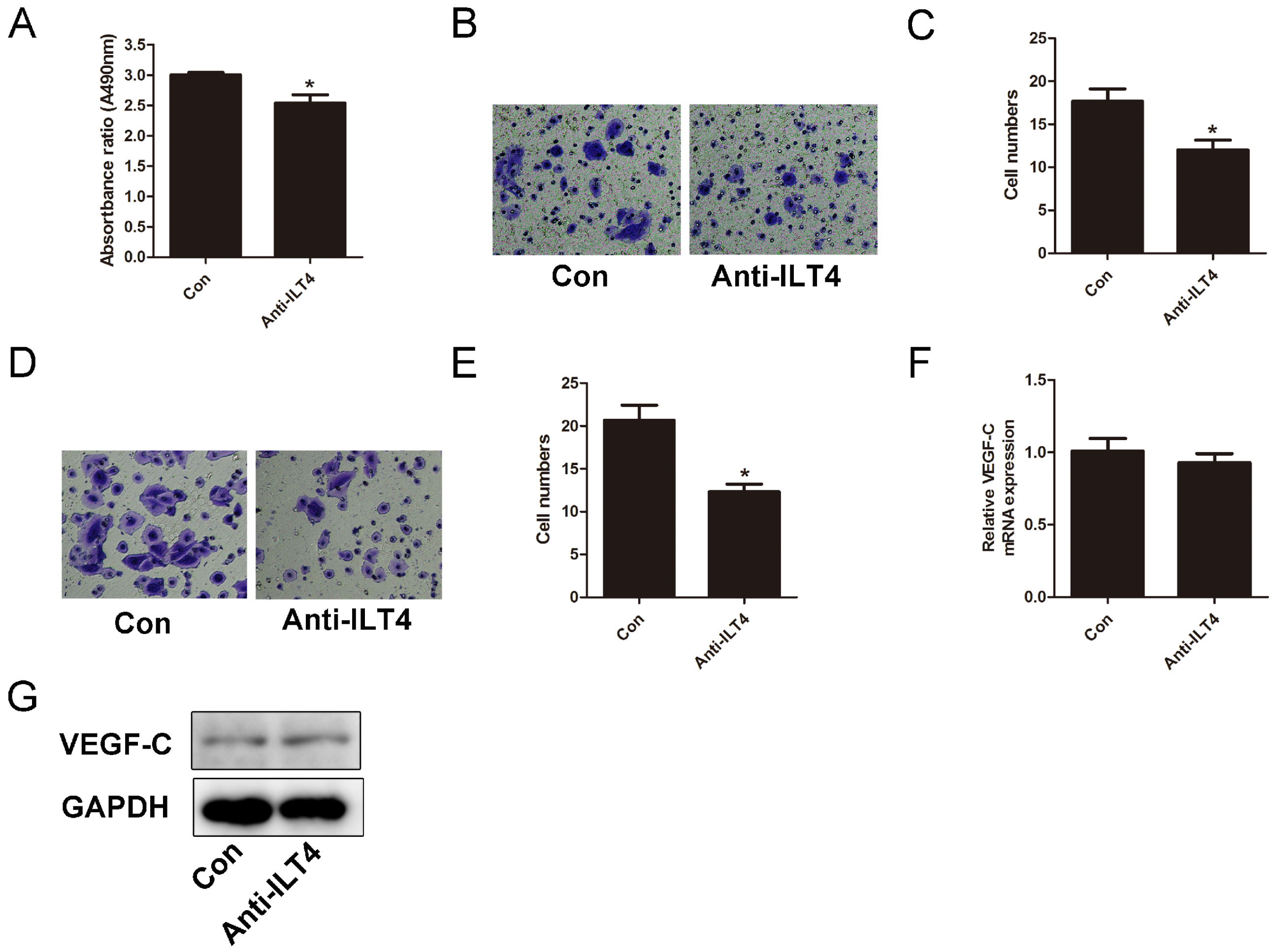

Supplementary Figure 8: The function of ILT4 blocking treatment on cell malignant phenotype and the expression of VEGF-C. A. Comparison of cell proliferation of A549 cells with or without ILT4 blocking antibody treatment by MTT assay. Comparison of **B.** and **C.** cell migration and **D.** and **E.** invasion of A549 cells with or without ILT4 blocking antibody by the invasion and migration assay, respectively. (Magnification × 400) The expression levels of VEGF-C in A549 cells with or without ILT4 blocking antibody treatment by **F.** PCR and **G.** Western blot analysis. The error bars indicate ± SEM. *$P < 0.05$; **$P < 0.01$ by Student's *t*-test. All the results were repeated thrice.

Supplementary Table 1: Correlation of ILT4 and VEGF-C in primary human NSCLC tissues and clinicopathological parameters

Variables	ILT4 expression			VEGF-C expression		
	Positive	Negative	*p*	Positive	Negative	*p*
Age (yr)						
< 60	13	32	0.044	20	25	0.955
≥ 60	29	31		27	33	
Gender						
Male	34	43	0.149	30	47	0.047
Female	8	20		17	11	
Smoking history(yr)						
< 30	8	23	0.055	17	14	0.179
≥ 30	34	40		30	44	
Histology						
Non-squamous NSCLC	21	40	0.17	19	34	0.064
Squamous NSCLC	21	23		28	24	
Cellular differentiation						
Well	16	37	0.038	26	27	0.372
Worse	26	26		21	31	
Primary tumor size(cm)						
< 5	30	41	0.496	39	32	0.002
≥ 5	12	22		8	26	
Regional lymph node involvement						
N0-N1	29	54	0.04	33	50	0.045
N2-N3	13	9		14	8	
2009 TNM stage groupings						
I–II	23	49	0.013	27	45	0.027
III	19	14		20	13	

Abbreviations: NSCLC, non-small cell lung cancer; TNM, tumor node metastasis.

Supplementary Table 2: Correlations of both ILT4 and VEGF-C co-expression with clinicopathological parameters in primary human NSCLC tissues

Variables	ILT4+/VEGF-C+	ILT4+/VEGF-C-		ILT4-/VEGF-C+		ILT4-/VEGF-C-	
	n	*n*	*p* 1	*n*	*p* 2	*n*	*p* 3
Age (yr)							
< 60	8	5	0.7	12	0.192	20	0.193
≥ 60	16	13		11		20	
Gender							
Male	19	15	1	11	0.025	32	1
Female	5	3		12		8	
Smoking history(yr)							
< 30	5	3	0.734	12	0.025	11	0.551
≥ 30	19	15		11		29	
Histology							
Non-squamous NSCLC	16	5	0.013	19	0.358	21	0.267
Squamous NSCLC	8	13		4		19	
Cellular differentiation							
Well	9	7	0.927	17	0.012	20	0.331
Worse	15	11		6		20	
Primary tumor size(cm)							
< 5	20	10	0.084	19	1	22	0.021
≥ 5	4	8		4		18	
Regional lymph node involvement							
N0-N1	14	15	0.162	19	0.134	35	0.008
N2-N3	10	3		4		5	
2009 TNM stage groupings							
I–II	11	12	0.179	16	0.1	33	0.002
III	13	6		7		7	

Note: *p* 1, *P* value between ILT4+/VEGF-C+ and ILT4+/VEGF-C-; *p* 2, *P* value between ILT4+/VEGF-C+ and ILT4-/VEGF-C+; *p* 3, *P* value between
ILT4+/VEGF-C+ and ILT4-/VEGF-C-.

Abbreviations: NSCLC, non-small cell lung cancer; TNM, tumor node metastasis.
